# Supplementary material for: PDK1 regulates VDJ recombination, cell-cycle exit and survival during B-cell development
Source: EMBO J. 2013 Mar 5;32(7):1008–22. doi: 10.1038/emboj.2013.40 (PMC3616287; doi:10.1038/emboj.2013.40)
Supplement: Supplementary Data [file emboj201340s1.pdf]

PDK1 regulates VDJ recombination, cell cycle exit and survival during B cell development.

Ram K. C. Venigalla, Victoria A. McGuire, Rosemary Clarke, Janet C. Patterson-Kane, Ayaz Najafov, Rachel Toth, Pierre C. McCarthy, Frederick Simeons, Laste Stojanovski and J. Simon C. Arthur.

Supplemental methods

Plasmids and retroviruses

Recombinant DNA procedures, restriction digests and ligations were performed using standard protocols. All PCR reactions were carried out using KOD Hot Start DNA polymerase (Novagen). DNA sequencing was performed by The Sequencing Service, College of Life Sciences, University of Dundee (www.dnaseq.co.uk).

cDNA coding for human Pax5 (NM_016734.1 GI:9951919) was amplified from ORF shuttle clone 100061597. The PCR product was cloned into vector pSC-B (Stratagene) and fully sequenced. Pax5 was sub-cloned from this vector as a BglII/Not1 insert into the BamH1/Not1 sites of expression vectors pcDNA5 FRT/TO.HA and pcDNA5 FRT/TO.GFP to generate plasmids for expression in mammalian cells of HA-Pax5 and GFP-Pax5 respectively. Pax5 was also sub-cloned into pGEX6P3 to generate a clone for bacterial expression of GST-tagged Pax5. Mutations S13I, A282T and A374P were made by PCR mutagenesis in pcDNA5 FRT/TO.GFP human Pax5 to generate a clone for expressing the mouse protein sequence with an N-terminal GFP tag. GFP-human Pax5 and GFP-mouse Pax5 cassettes were re-amplified, cloned into pSC-B and then subcloned into the retroviral expression plasmid pLZRSpBMN as HindIII/Not1 inserts. Human Pax5 was also sub-cloned into a pLZRSpBMN plasmid that contained the dsRed monomer sequence as a HindIII/Not1 insert to generate a plasmid for retroviral expression of dsRed-Pax5.

cDNA coding for mouse Bcl2-A1b (NM_007534) was amplified from IMAGE EST clone 40091848. The PCR product was cloned into vector pSC-B (Stratagene) and fully sequenced. Bcl2-A1b was sub-cloned from this vector as a BamH1/BamH1 insert into the BglII/BamH1 sites of expression vector pEGFP-C1 for mammalian expression of GFP-Bcl2-A1b. Bcl2-A1b was re-amplified from this plasmid and cloned as a HindIII/Not1 insert into pLZRSpBMN plasmids containing GFP, mCherry or dsRed-monomer coding sequences, to generate plasmids for retroviral expression of GFP-Bcl2-A1b, mCherry-Bcl2-A1b and dsRed-Bcl2-A1b respectively.

To generate virus, plasmids were transfected into Phoenix (293T) cells using a calcium phosphate method and retrovirus was collected from 48 hours for 3 days and was concentrated by spinning at 20000xg for 4 hours at 4^0C.

Supplementary table 1: Antibodies

Antibody	Clone	Supplier	Use
anti-IgM	II/41	BD Biosciences	FACS
anti-SCF-1	E13-161.7	BD Biosciences	FACS
anti-IgG1	A85-1	BD Biosciences	FACS
anti-IgG2a/b	R2-40	BD Biosciences	FACS
anti-BP1	6C3	BD Biosciences	FACS
anti-IgD	11-26	BD Biosciences	FACS
anti-CD43	S7	BD Biosciences	FACS
anti-CD3e	145-2C11	BD Biosciences	FACS
anti-CD45R	RA3-6B2	BD Biosciences	FACS
anti-CD11b	M1/70	BD Biosciences	FACS
anti-CD19	ID3	BD Biosciences	FACS
anti-CD117	2B8	BD Biosciences	FACS
anti-Ter119	Ter-119	BD Biosciences	FACS
anti-Gr1	RB6-8C5	BD Biosciences	FACS
anti-pan-NK	DX5	BD Biosciences	FACS
anti-λ5	LM34	BD Biosciences	FACS
anti-Vpreb or CD179a	R3/Vpreb	BD Biosciences	FACS
anti-CD45RA	14.8	BD Biosciences	FACS
anti-CD11c	HL3	BD Biosciences	FACS
anti-p27	57	BD Biosciences	FACS
anti-Ccnd3	1	BD Biosciences	FACS
anti-BLNK	2B11	BD Biosciences	FACS
anti-TCRγδ	GL3	BD Biosciences	FACS
anti-CD4	RM4-5	BD Biosciences	FACS
anti-CD8	53-6.7	BD Biosciences	FACS
anti-Thy1.2	53-2.1	BD Biosciences	FACS
anti-CD25	PC65	BD Biosciences	FACS
anti-TCRb	H57-597	BD Biosciences	FACS
anti-CD45.1	A20	BD Biosciences	FACS
anti-CD45.2	104	BD Biosciences	FACS
anti-CD44	IM7	BD Biosciences	FACS
anti-streptavidin		BD Biosciences	FACS
anti-pre-BCR	SL156	BD Biosciences	FACS
anti-CD127	A7R34	eBiosciences	FACS
anti-IRF4	3E4	eBiosciences	FACS
anti-IRF8,		eBiosciences	FACS
anti-Aiolos	8B2	eBiosciences	FACS
anti-Pax5	1H9	eBiosciences	FACS
anti-Ikaros	2A9	Active Motif	FACS
anti-BCL2A1	EP517Y	Epitomics	FACS
anti-rabbit IgG FITC		Santa Cruz Biotechnology	FACS
p-T308 Akt1	4056	Cell Signalling Technology	immunoblotting
p-S471 Akt1	9271	Cell Signalling Technology	Immunoblotting
p-S235/236 S6 ribosomal protein	4856	Cell Signalling Technology	immunoblotting
S6 ribosomal protein	2217	Cell Signalling Technology	Immunoblotting
p-T202/Y204 ERK1/2	9101	Cell Signalling Technology	immunoblotting
ERK1/2	9102	Cell Signalling Technology	Immunoblotting
p-T24/32 FOXO1/3	9464	Cell Signalling Technology	immunoblotting
FOXO1	2880	Cell Signalling Technology	Immunoblotting
p-S227 RSK2	Sc-12445-R	Santa Cruz Biotechnology	immunoblotting
Akt1	Antibody raised against residues 466-480 of human Akt1	In house	Immunoblotting
Annexin V		BD Biosciences	FACS

Supplementary table 2: qPCR primer sets

Gene	Sense primer	Antisense primer
Pax5	GAGTCTGTGACAATGACACTGTGC	AGCCTGTAGACACTATGCTGTGAC
BCL2A1	GATTGCCCTGGATGTATGTGCTTAC	AGCCATCTTCCCAACCTCCATTC
Irf4	GGACTACAATCGTGAGGAGGAC	ACGTCACAGGACATTGATATGG
Irf8	GGGGCTGCCTAAGTTGTATG	CTCGGATGAACTGGTTGGTG
p27	GTGTCCAGGGATGAGGAAG	CGGAGCTGTTTACGTCTGG
cmyc	ATCATCCAGGACTGTATGTGGAG	TTCTTGCTCTTCTTCAGAGTCG
lamda5	GCGGAATTCTCAGCAGAAAGGAGCAGAGCT	GCGAAGCTTACACACTACGTGTGGCCTTGT
vpreb	ATGCTGCTGGCCTATCTCACAGG	ATGGTCGTTGCTCAGGGTACAGG
PDK1	AGATGTGATGTCACGCCTGGATC	CACAATCTCAGCCGTGTAAAACCG
Aiolos	GACACGTGCCCTATGACAACAGCAG	GCATGCGTAGTTGCAGAGGTGACAC
Ikaros	AAGTCTGTGTCATCGGAGCGAGAGG	CATCCTGCGAGTTCTCTGAGGC
Rag1	GTGGAGCAAGGTAGCTTAGCCAAC	TTTCATCGGGTGCAGAACTGAAGC
Rag2	AAACAGTGACTCTTCCCCAAGTGC	ATTTTGGCACTGAAGGCCAGAGG
LigaseIV	TCTGCCTTTAAGCCAATGCT	GTGAGAGAGCCTTCCTGTGG
Artemis	GATGCACTGTGTCCACAAGAT	TTCCTGCCACTCAGGGTGT
DNA-PKc	AAACCTGTTCCGAGCTTTTCTG	TCTCAATCTGAGGACGAATTGC
Ku80	ATGGCGTGGTCGGTAAATAAG	CCTGTCGTTGGACAAACATAGTC
Ku70	ATGTCAGAGTGGGAGTCCTAC	TCGCTGCTTATGATCTTACTGGT
TdT	AGAGACCTTCGGCGCTATG	TGACAGTCTTCCCCTTAGTCC
XRCC4	CTGGAGGAGAGTACCAAACCT	CTGGGGTAGTGAAGAGGCAAG
E47	GGGAGGAGAAAGAGGATGA	CCGGTCCCTCAGGTCCTTC
Iga	CCTGCCTCTCCTCCTCTTCTTGTC	GACTGAAGGCTGAACCACCATGTG
Igb	GTGCCCATCTTCCTGCTACTTGAC	TGCTCTCCTACCGACCACTTTACC
IL7r	CCAGCAAGGGGTGAAAGCAACTG	CACTCGCTCCAGAAGCCTTTGAAG
Igk GLT	GAGGGGGTTAAGCTTTCGCCTACCCAC	GTTATGTCGTTCATACTCGTCCTTGGT CAA
Vk-JCK	GGCTGCAGCTTCAGTGGCAGTGGATCAGGAAC	TTGGTCAACGTGAGGGTGCTG
VH7183-DJCμ	CGGTACCAAGAACAACCTGTACCTGCAAATGACC	ATGCAGATCTCTGTTTTTGCCTCC
VHJ558-DJCμ	CGAGCTCTCCAACACAGCCTACATGCAACTCAAC	ATGCAGATCTCTGTTTTTGCCTCC
Vλ-Jcλ1	CGCGAATTCTCAGGCTCCCTGATTGGAGACAAGG	GACCTAGGAACAGTCAGCACGGG
MCL1	AGGACGAAACGGGACTGGCTTG	AGCACATTTCTGATGCCGCCTTC
HPRT	GGGGGCTATAAGTTCTTTGCTGACC	TCCAACACTTCGAGAGGTCCTTTTCAC
Igλ GLT	CTTGAGAATAAAATGCATGCAAGG	TGATGGCGAAGACTTGGGATGG
Vh7183 GLT	CGGTACCAAGAACAACCTGTACCTGCAAATGACC	GTCTCTCCGCGCCCCCTGCTGGTCC
VHJ558 GLT	ACCATGGGATGGAGATGGATCTTTC	CTCAGGATGTGGTTACAACACTGTG

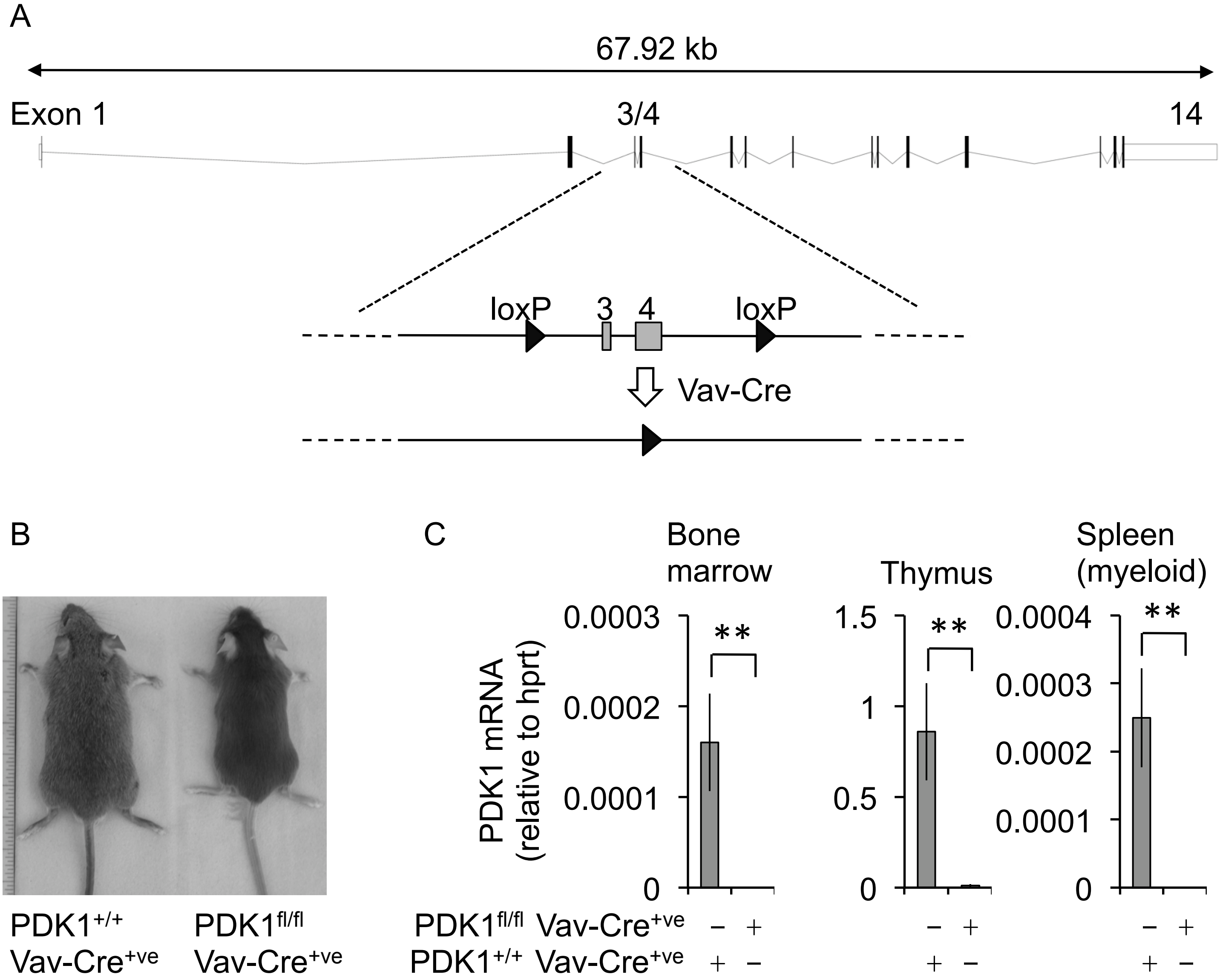

Supplementary Figure 1. Generation of hematopoietic PDK1 knockout mice.

To generate mice with a knockout of PDK1 in blood cells, mice with loxP sites either side of exons 3 and 4 ($PDK1^{fl/fl}$) were used (A). Deletion of exons 3 and 4 results in the loss of the coding sequence from amino acid 99 to 156, which encodes the 1st half of the kinase domain and also introduces a frame shift mutation. To generate the conditional deletion mice were crossed to a Vav-Cre transgenic line, which deletes early during hematopoietic development in the bone marrow. As Vav-Cre may result in some deletion in the male germline female $PDK1^{+/fl}$/Vav-Cre^{+ve} mice were used for breeding. $PDK1^{fl/fl}$/Vav-Cre^{+ve} mice tended to be smaller that littermate controls (B). To confirm that PDK1 had been deleted, total RNA was isolated from the bone marrow, thymus and myeloid cell fraction from the spleen from $PDK1^{+/+}$/Vav-Cre^{+ve} and $PDK1^{fl/fl}$/Vav-Cre^{+ve} mice. PDK1 mRNA levels were determined by qPCR using primers that bound in the deleted region. ** represents a p value (students ttest) of <0.01.

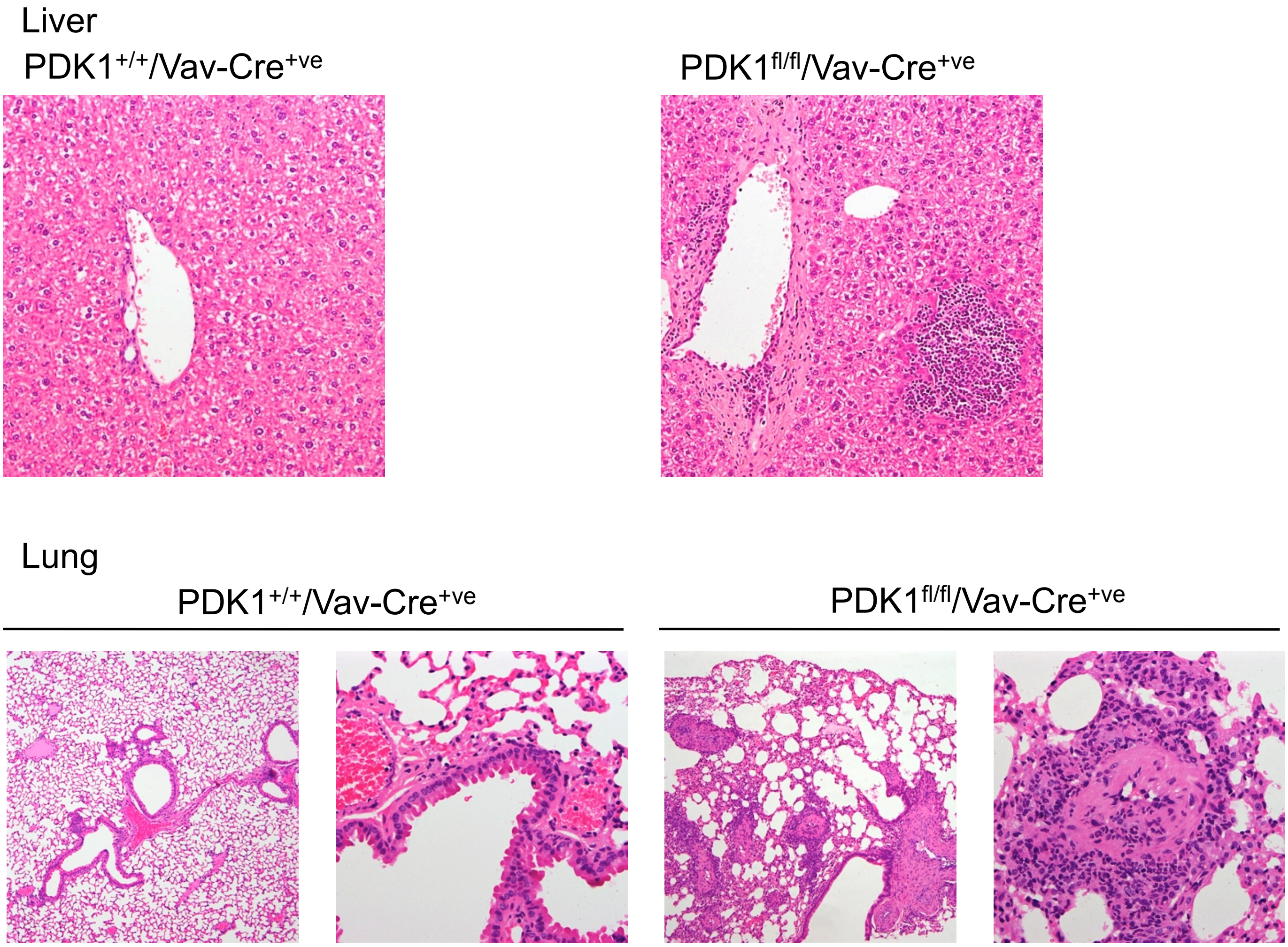

Supplementary Figure 2. Immune cell recruitment to the lungs and liver of PDK1 knockout mice.

In the livers of PDK1$^{fl/fl}$Vav-Cre^{+ve} mice myeloid cells were increased in number in sites where hematopoietic cells are frequently noted, including portal tracts and sinusoids; occasional nodular clusters of cells in the latter location were expanding the sinusoids and compressing adjacent hepatocytes. In the lung tissue, myeloid cells at various stages of maturity were surrounding and infiltrating walls of blood vessels (both arterial and venous) and extending into contiguous alveolar septa. The arterial walls were prominent due to marked muscular hypertrophy.

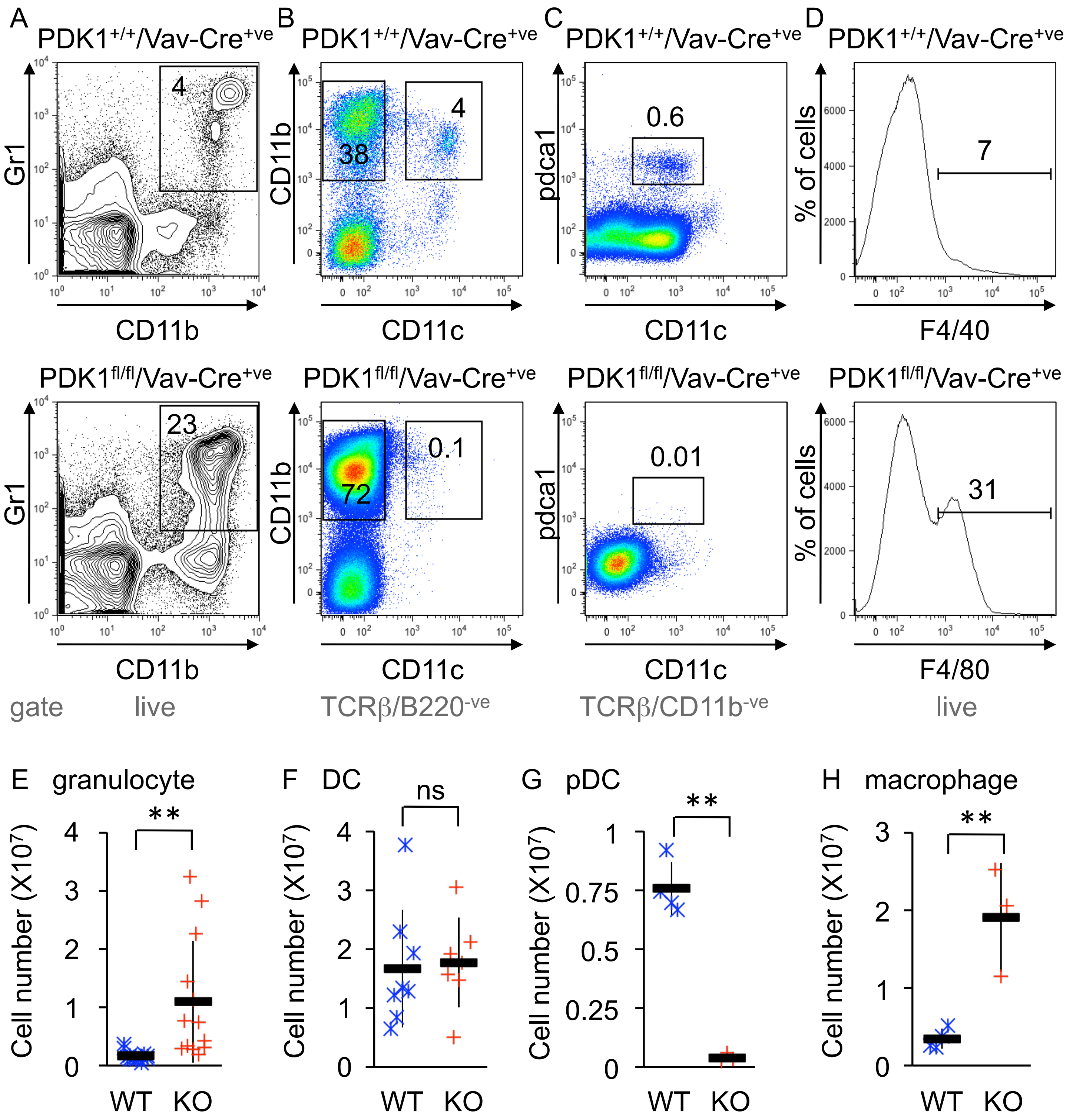

Supplementary Figure 3. PDK1 knockout does not block myeloid cell development.
The populations of myeloid cells in the spleens of PDK1$^{+/+}$/Vav-Cre^{+ve} and PDK1$^{fl/fl}$/Vav-Cre^{+ve} mice were analyzed by FACS (A-D), and used to calculate the absolute numbers of the different myeloid cells in the spleen (E-H). Loss of PDK1 resulted in increased numbers of granulocytes (A and E) and macrophages (D and H). Although there was a decrease in the percentage of the PDK1$^{fl/fl}$/Vav-Cre^{+ve} myeloid dendtic cells (mDC) when gated on TCRβ/B220-ve cells (B), absolute numbers were similar due to the increased numbers of TCRβ/B220 negative cells in the PDK1$^{fl/fl}$/Vav-Cre^{+ve} spleens (F, Fig 1). In contrast to other myeloid cells, the numbers of plasmacytoid dendritic (pDC) cells was decreased in the the PDK1$^{fl/fl}$/Vav-Cre^{+ve} spleens (C and G). ** represents a p value (students ttest) of <0.01; ns represents a p valuse of >0.05.

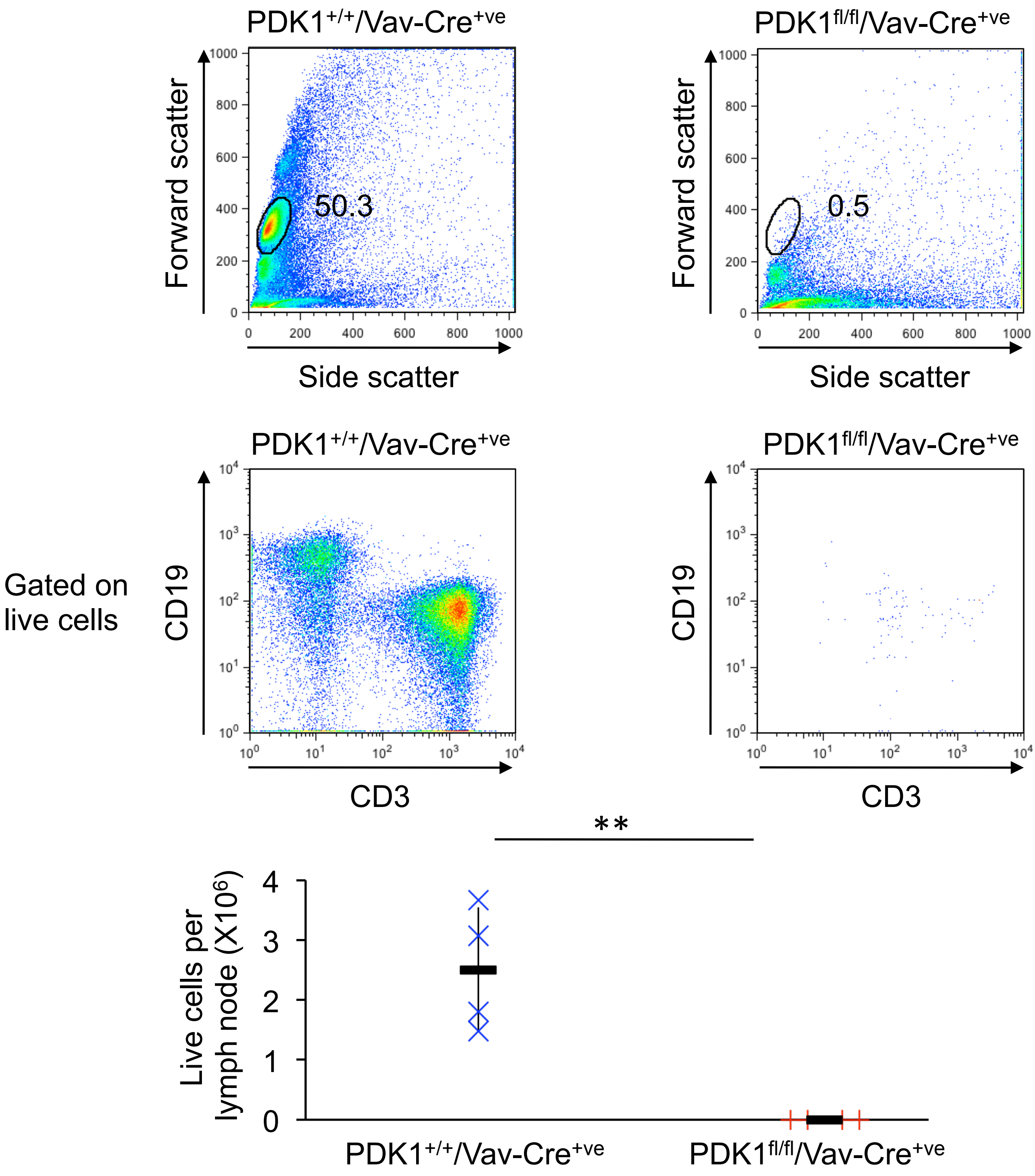

Supplementary Figure 4. PDK1 knockout mice lack T or B cells in their lymph nodes. Cells were isolated from PDK1$^{+/+}$/Vav-Cre^{+ve} and PDK1$^{fl/fl}$/Vav-Cre^{+ve} lymph nodes and stained for CD19 to identify B cells and CD3 for T cells. While clear T and B cell populations were present in PDK1$^{+/+}$/Vav-Cre^{+ve} lymph nodes, very few live cells were obtained from PDK1$^{fl/fl}$/Vav-Cre^{+ve} lymph nodes and no clear T or B cells populations could be identified. Cell numbers were determined on a FACSVerse using DAPI to exclude dead cells; error bars represent the standard deviation while the p value (student t-test) between wild type and knockout cells was 0.0029.

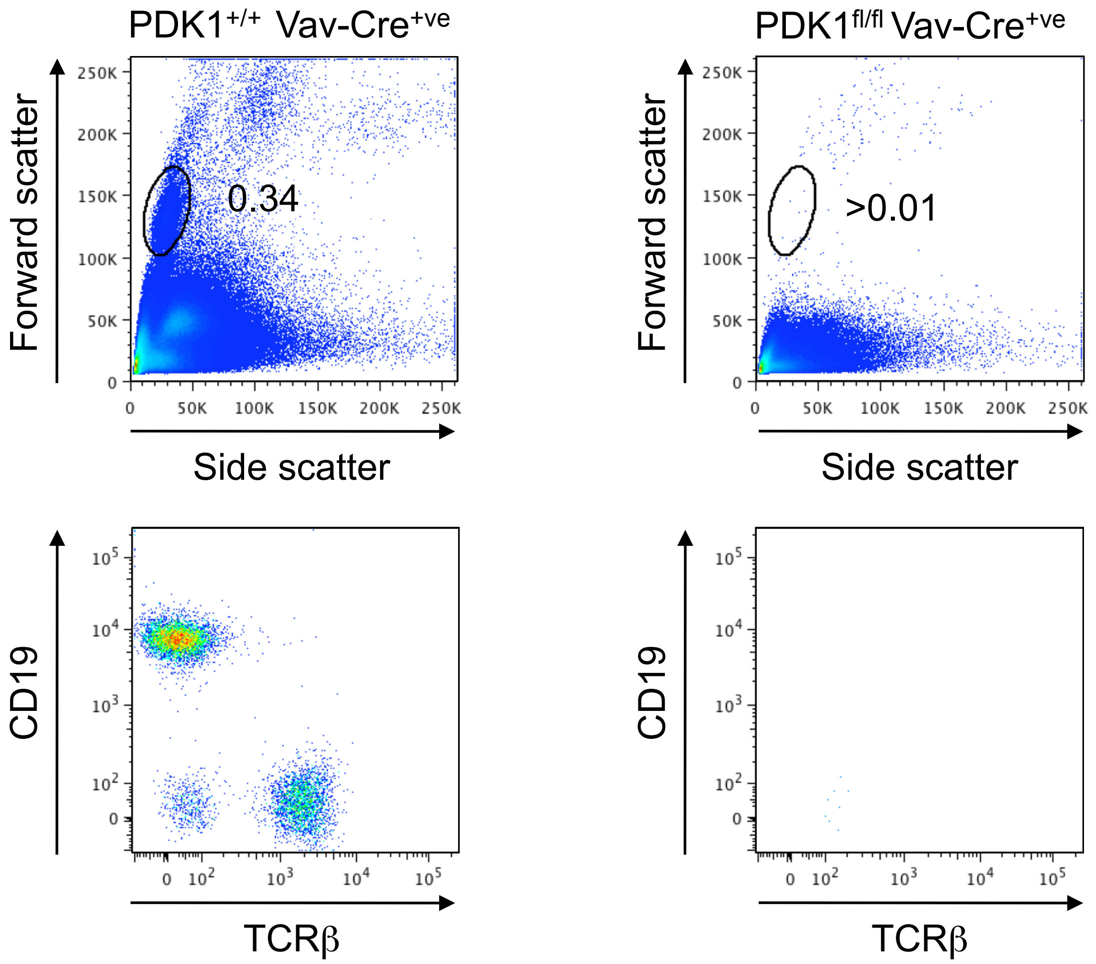

Supplementary Figure 5. PDK1 knockout mice lack circulating T and B cells.
Blood was taken from PDK1$^{+/+}$Vav-Cre^{+ve} and PDK1$^{fl/fl}$Vav-Cre^{+ve} mice and, following red blood cell lysis, stained for CD19 to identify B cells and TCRβ for T cells. While clear T and B cell populations were present in PDK1$^{+/+}$Vav-Cre^{+ve} blood, very few lymphocytes could be detected based on forward and side scatter (upper panels, black oval), and no CD19 or TCRβ positive cells were observed in this gate in the PDK1$^{fl/fl}$Vav-Cre^{+ve} blood (lower panels).

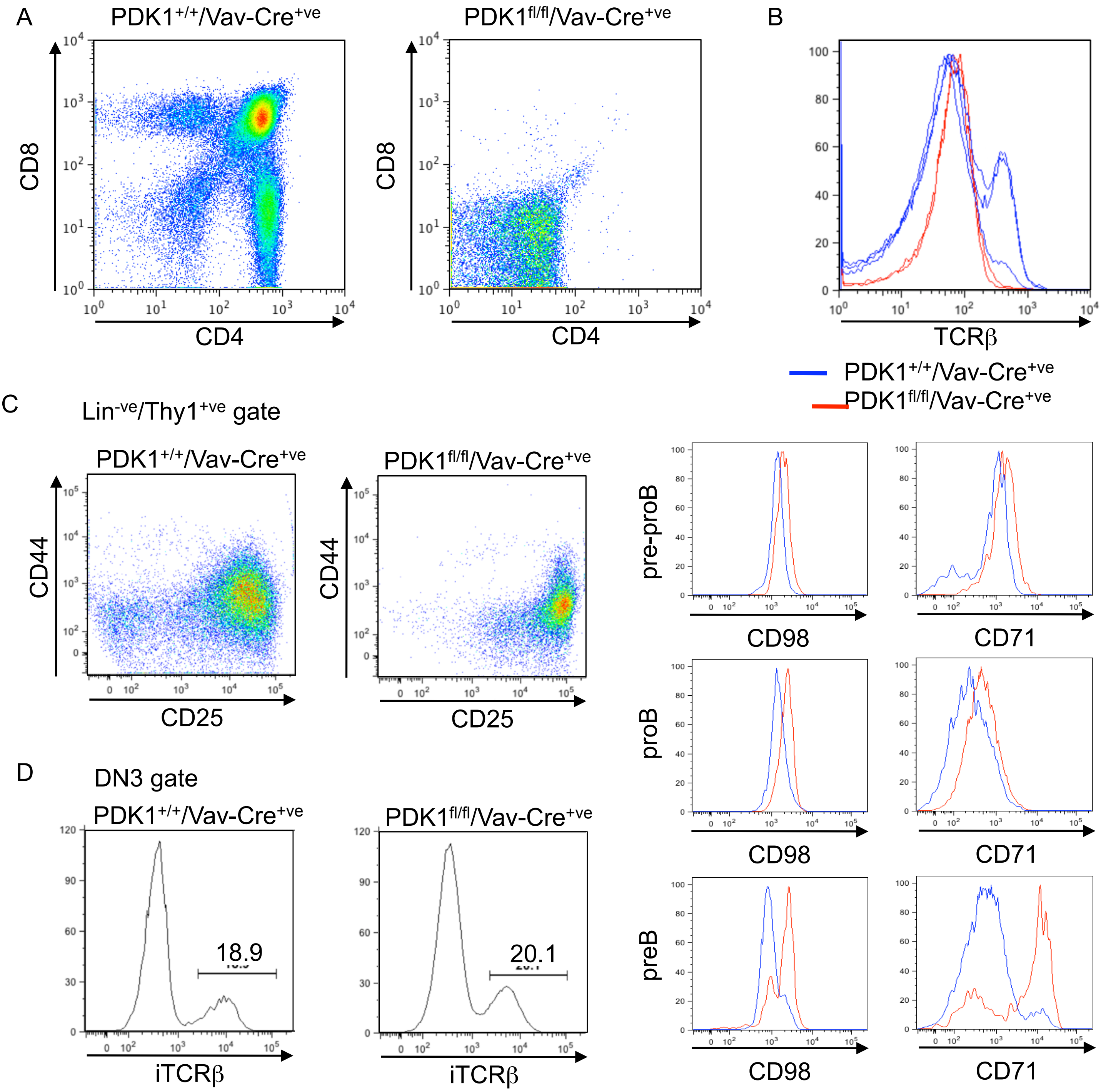

Supplementary Figure 6. PDK1 knockout arrests T cell development at the DN3 stage of development.

Thy1^{+ve} Thymocytes from PDK1$^{+/+}$/Vav-Cre^{+ve} and PDK1$^{fl/fl}$/Vav-Cre^{+ve} mice were stained for CD4 and CD8 (A), revealing that the loss of PDK1 blocked the formation of DP cells. In agreement with this, knockout of PDK1 prevented the upregulation of TCRβ on the cell surface (B). To examine this further the development of DN cells was examined by analyzing CD44 and CD25 staining in Lin^{-ve} Thy1^{+ve}cells cells (C). This demonstrated that PDK1 was required for the development of T cells past the DN3 stage. Intracellular staining for TCRβ in DN3 cells (D) showed that PDK1 knockout did not block TCRβ rearrangement and expression of the preTCR. (E) Bone marrow was isolated from PDK1$^{+/+}$/Vav-Cre^{+ve} and PDK1$^{fl/fl}$/Vav-Cre^{+ve} mice, stained for CD98 and CD71 in addition to the appropriate B cell markers and analyzed by FACS. Loss of PDK1 resulted in increased CD98 and CD71 expression in pre-pro (B220^{+ve}/IgM^{-ve}/CD43^{+ve}CD19^{-ve}), pro (B220^{+ve}/IgM^{-ve}/CD43^{+ve}CD19^{+ve}) and preB (B220^{+ve}/IgM^{-ve}/CD43^{-ve}CD19^{+ve}) cells.

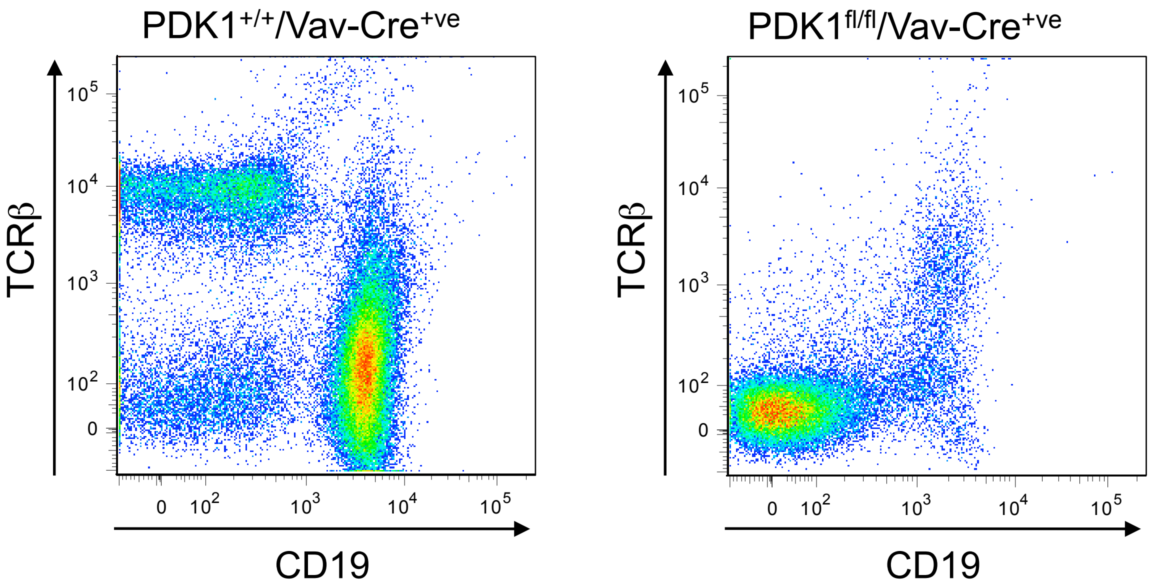

Supplementary Figure 7. Bone marrow progenitors from PDK1 knockout mice do not efficiently re-colonize the T and B cell populations in Rag2 knockout mice.
Bone marrow was isolated from either PDK1+/+/Vav-Cre+ve or PDK1fl/fl/Vav-Cre+ve mice and depleted of B220+ cells as described in the methods. Cells were then injected into sub-lethally irradiated Rag2 knockout mice, and 9 weeks later mice were sacrificed. T and B cells were identified by FACS analysis of splenocytes stained for TCRβ and CD19.

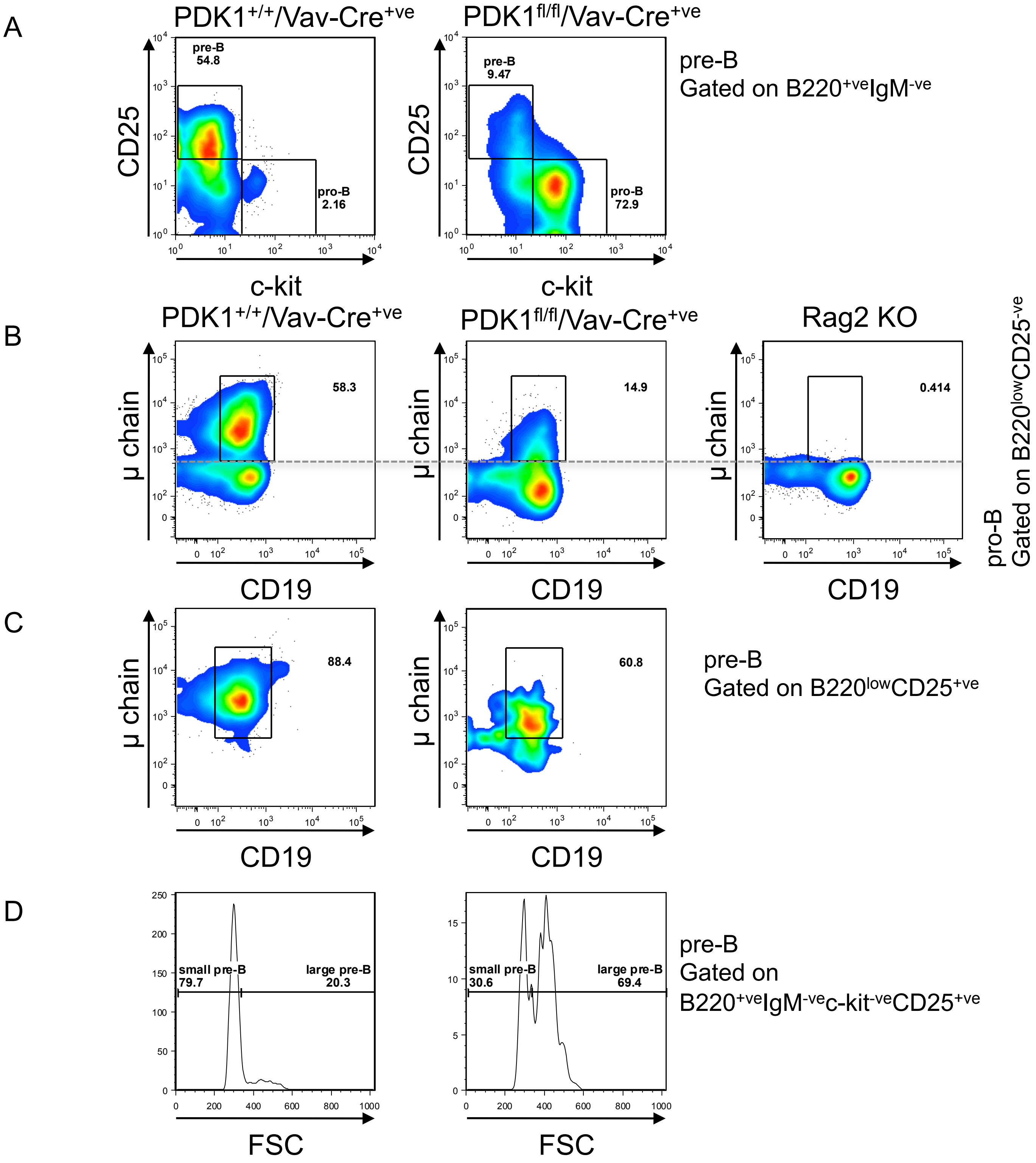

Supplementary Figure 8. Analysis of pre-B and pro-B cells.

Bone marrow was isolated from the indicated mice and stained with B220, IgM, c-Kit, CD25 and/or the intracellular heavy chain. (A) Cells were classified into pro-B ($B220^{+ve}IgM^{-ve}c\text{-}kit^{+ve}CD25^{-ve}$) and pre-B ($B220^{+ve}IgM^{-ve}c\text{-}kit^{-ve}CD25^{+ve}$). The levels of intracellular heavy chain in the pro-B (B) and pre-B (C) cells are shown. Rag2 knockouts were analysed to allow levels of background staining to be determined in cells with no heavy chain. The pre-B cells were also classified into small pre-B and large pre-B based on forward side scatter (D).

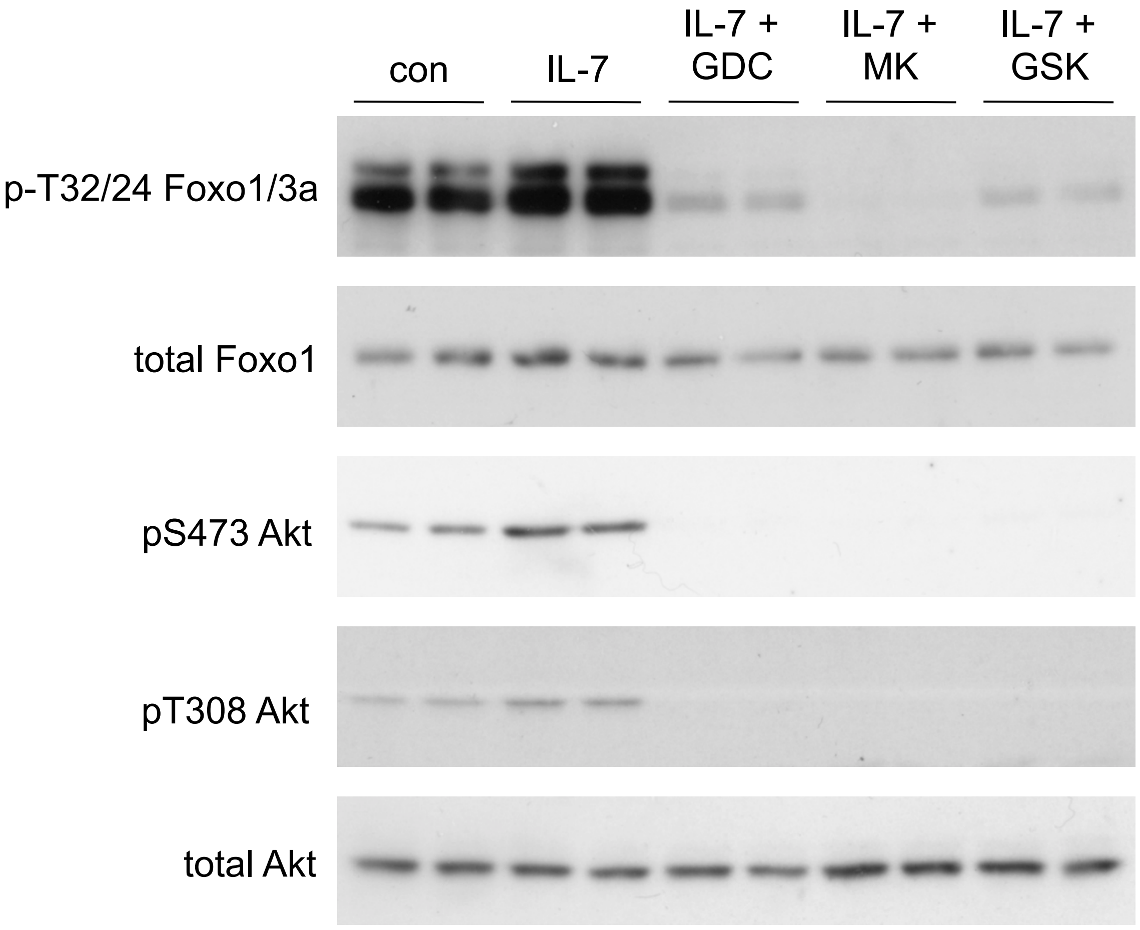

Supplementary Figure 9. PDK1 regulates Akt activation by IL-7 in the 70z/3 pre-B cell line. 70z/3 cells were incubated where indicated with 1 μM GDC-0941 μM MK2206 or 3 μM GSK2334470 for 1h. Cells were then stimulated with 20 ng/ml IL-7 for 15 min and the levels of the indicated proteins determined by immunoblotting. GDC-0941 is a specific inhibitor of PI3-K, MK2206 an inhibitor of Akt and GSK2334470 an inhibitor of PDK1.

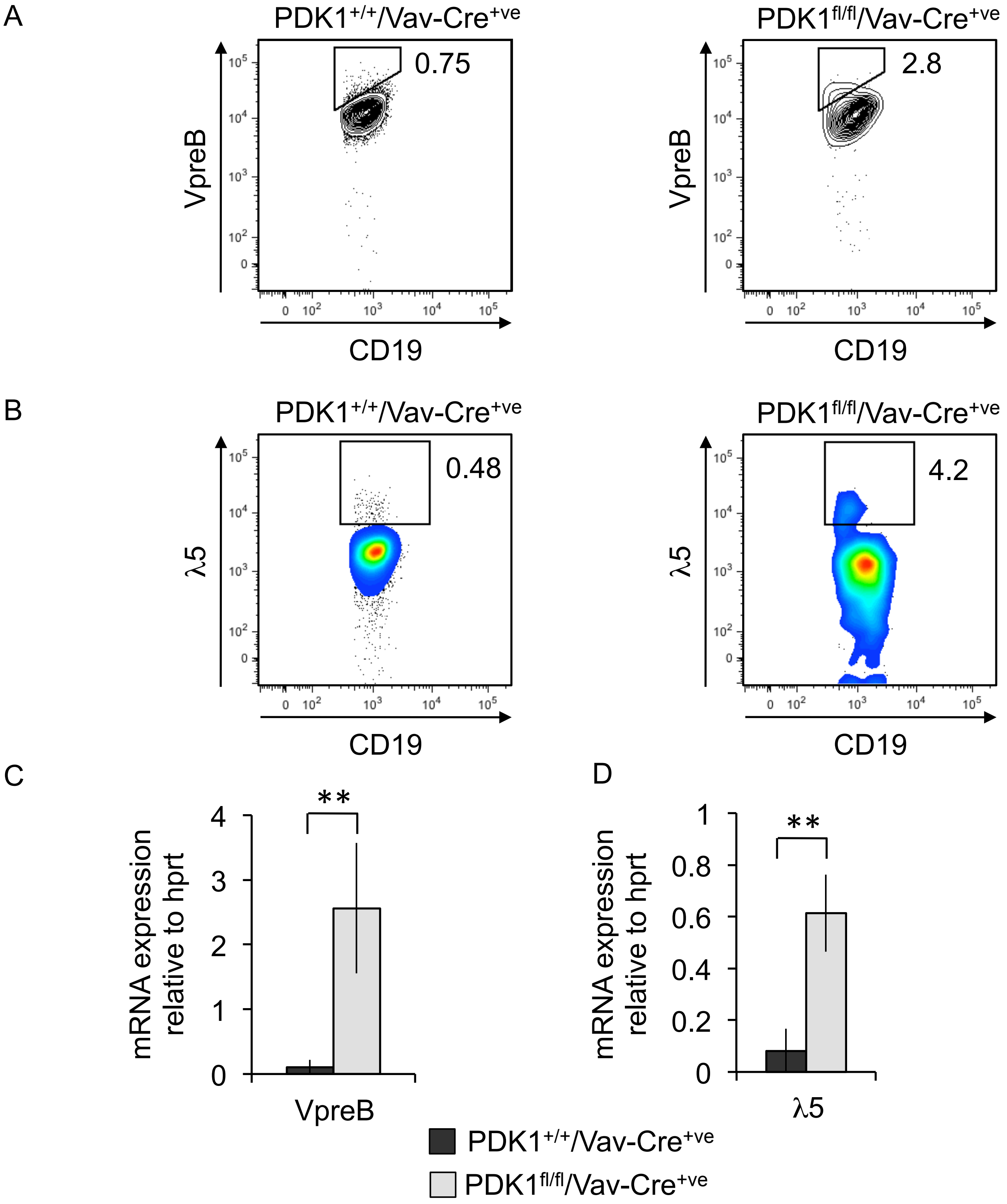

Supplementary Figure 10. Expression of preBCR components in pre-B cells.
Bone marrow was isolated *ex vivo* from PDK1$^{+/+}$/Vav-Cre^{+ve} or PDK1$^{fl/fl}$/Vav-Cre^{+ve} mice, and the levels of VpreB and λ5 examined by FACS. Gating on CD19^{+ve}B220^{+ve}IgM^{-ve}CD43^{-ve} cells was used to restrict the analysis to pre B cells for VpreB (A) and λ5 (B) staining. Alternatively CD19^{+ve}B220^{+ve}IgM^{-ve}CD43^{-ve} DAPI^{-ve} pre-B cells were isolated by FACS sorting, and the levels of VpreB (C) and λ5 (D) mRNA determined by qPCR. Error bars represent the standard deviation of RNA from FACS sorted pre-B cells of 3 independent pools of mice per genotype, $p<0.01$ (students t-test) is indicated by **.

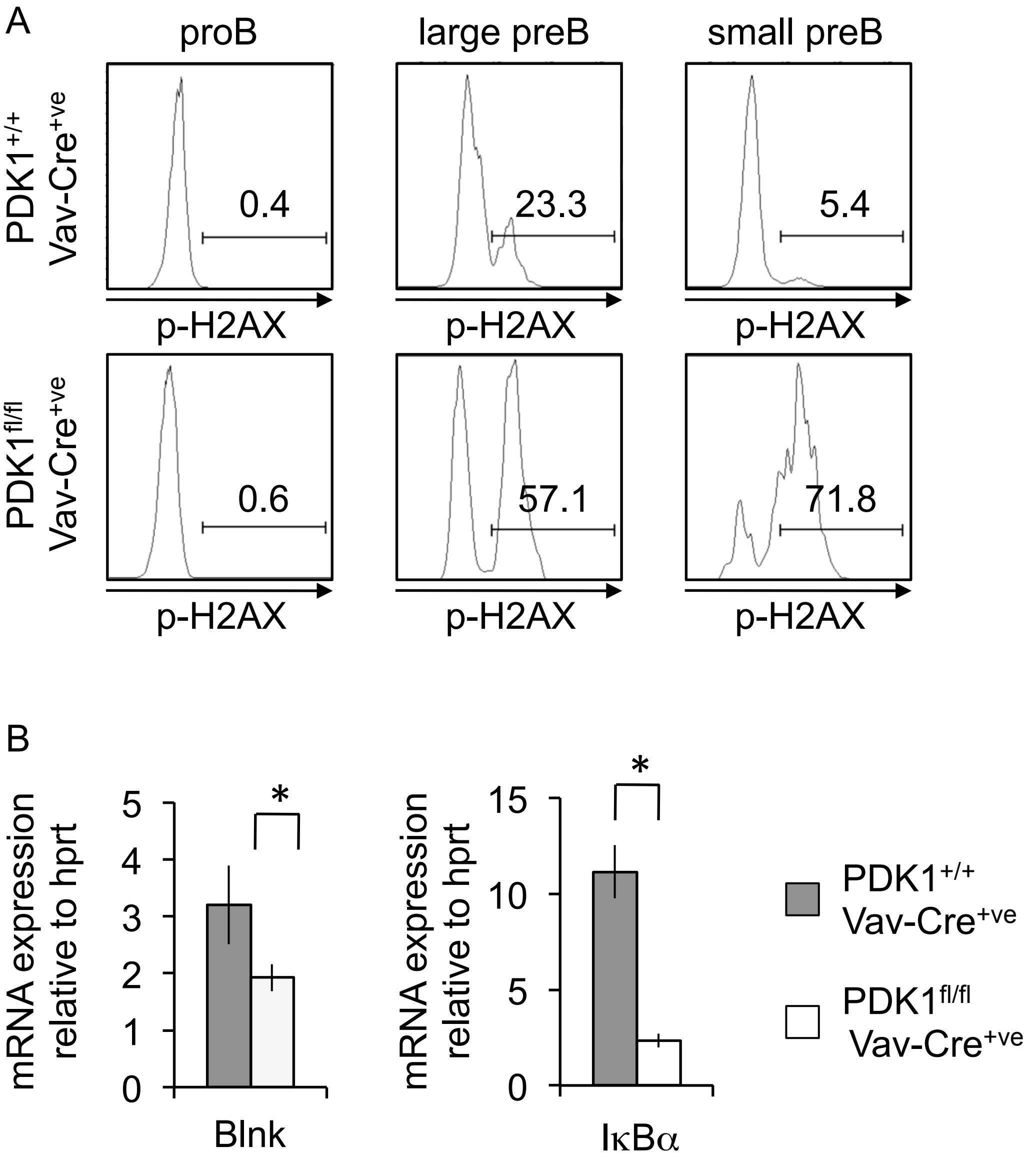

Supplementary Figure 11. PDK1 promotes cell survival in preB cells.
The levels phospho-H2AX in *ex vivo* PDK1$^{+/+}$/Vav-Cre^{+ve} and PDK1$^{fl/fl}$/Vav-Cre^{+ve} cells was determined by FACS staining of pro-B (CD19^{+ve}IgM^{-ve}CD43^{high}B220^{+ve}), large pre-B (FSC^{high}CD19^{+ve}IgM^{-ve}CD43^{-ve}B220^{+ve}) or small preB (FSC^{low}CD19^{+ve}IgM^{-ve}CD43^{-ve}B220^{+ve}) cells (A). Histone H2AX phosphorylation occurs at double strand breaks in genomic DNA and is therefore strongly upregulated when DNA is fragmented during apoptosis. qPCR was used to determined the mRNA levels of Blnk and IκBα in FACS sorted pre-B cells from PDK1$^{+/+}$/Vav-Cre^{+ve} and PDK1$^{fl/fl}$/Vav-Cre^{+ve} mice (B). Error bars represent the standard deviation of 3 independent preparations of cells per genotype. A p value (students ttest) of less than 0.05 is indicated by *.

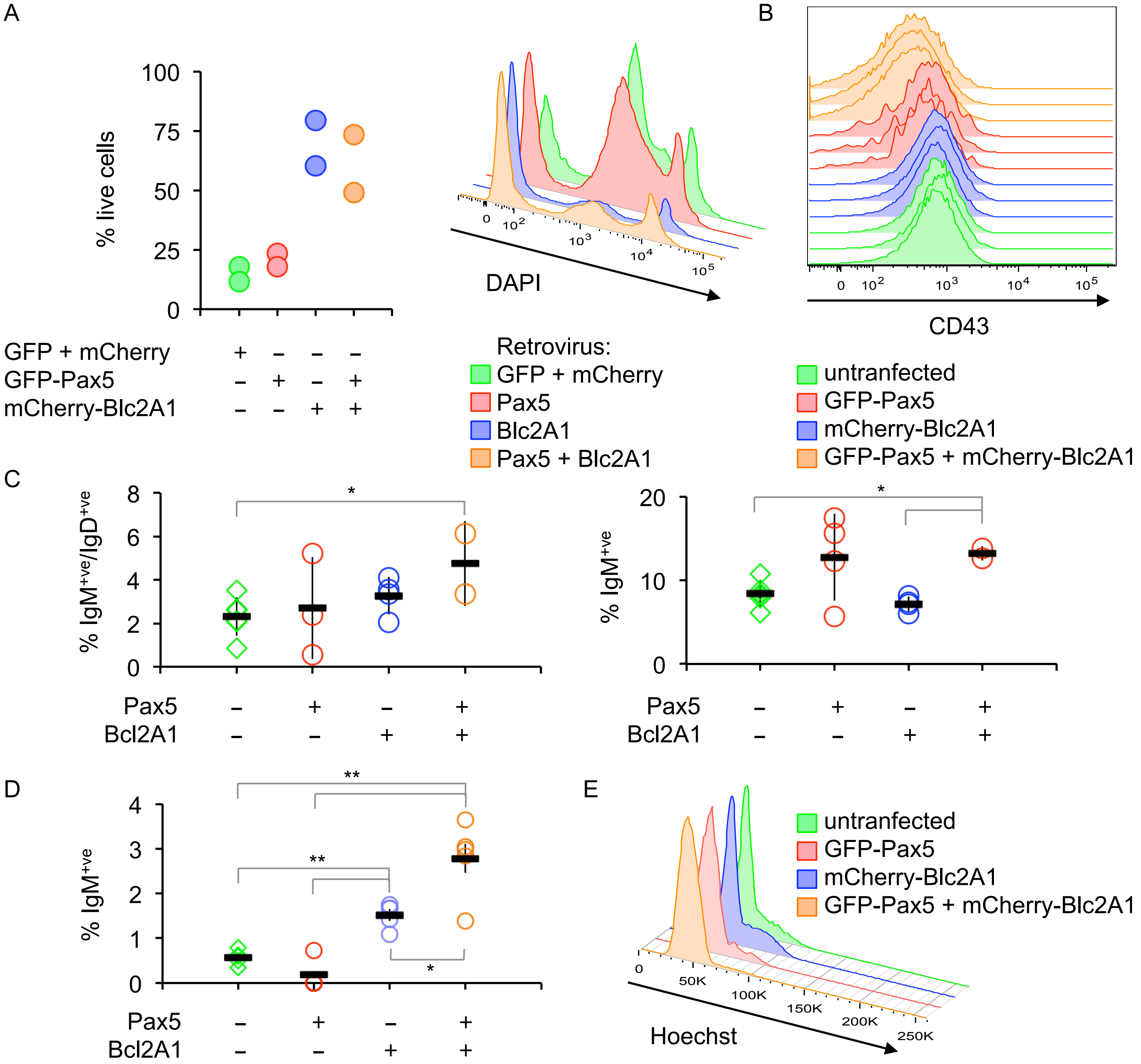

Supplementary figure 12. Rescue of B cell development by Pax5 and Bcl2A1.

(A) PDK1 knockout pro-B cells were isolated and transfected with the retroviruses encoding the indicated proteins as described in the methods. Cells were maintained in IL-7 for 4 days and the IL-7 withdrawn for 2 days and cells isolated and the % of live cells determined by DAPI staining. Representative histograms are shown on the left and the % live cells from two independent experiments on the right. (B) PDK1 knockout pro-B cells were transfected with a combination of retroviruses encoding GFP-Pax5and mCherry-Bcl2A1 as described in the methods. After 4 days in culture with IL-7, the IL-7 was withdrawn for 2 days and the cells analyzed by FACS. Cell were gated for expression GFP-Pax5 and mCherry-Bcl2A1 and the expression of CD43 in live B220+veCD19+ve cells determined. Results are shown for three independent transfections. (C) Wild type pro-B cells were isolated and transfected with viruses encoding GFP-Pax5 and/or mCherry-Bcl2A1 and maintained for 4 days in IL-7 followed by 2 days without IL-7. The % of IgM and IgD positive cells was determined after gating for expression of GFP-Pax5 or mCherry-Bcl2A1 as indicated. (D) The data for the mCherry-Bcl2A1 and GFP-Pax5 transfected cells form figure 8C and E was analyzed for the expression of IgM in cells expressing the indicated proteins. (E) Representative Hoechst stains for the cell cycle analysis described in figure 8G in transfected PDK1 knockout pro-B cells are shown. Error bars represent standard deviation and ** and * indicate $P<0.01$ and 0.05 respectively.

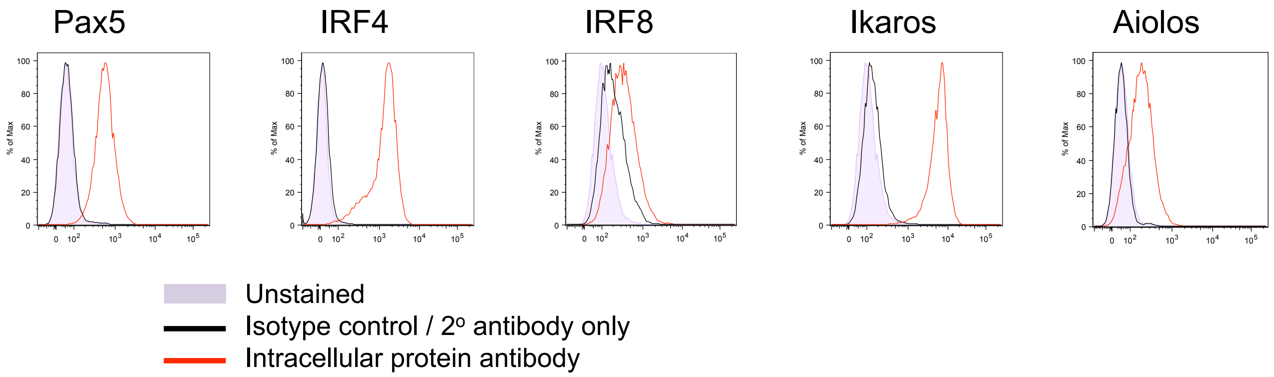

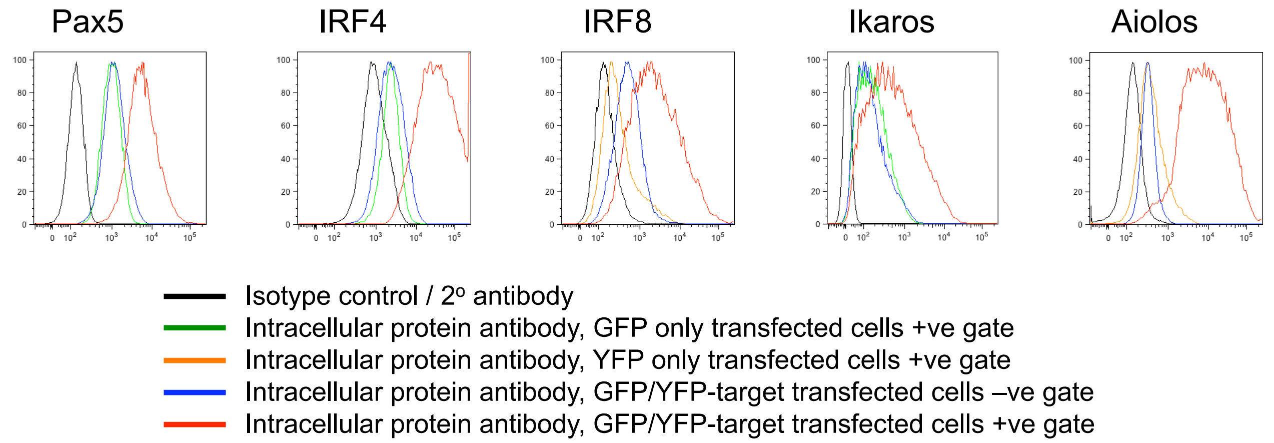

protein	Clone	Flurophore	Cat Number	Manufacturer	Isotype / secondary	Clone	Flurophore	Cat Number	Manufacturer
Pax5	1H9	PE	12-9918-80	eBioscience	Rat IgG2a	eBR2a	PE	12-4321-81	eBioscience
Aiolos	8B2	PE	12-5789-80	eBioscience	Mouse IgG1	P3	PE	12-4714-71	eBioscience
IRF4	3E4	Alexa Fluor 647	51-9858-80	eBioscience	Rat IgG1	eBRG1	Alexa Fluor 647	51-4301-80	eBioscience
Ikaros	2A9	--	39355	Active Motif	MouseIgG1	A85-1	APC	530089	BD Biosciences
sIRF8	N/A	--	15-5850-82	eBioscience	Rabbit IgG1	N/A	APC	4050-11S	Southern Biotech

Supplementary Figure 13. Isotope control data for intracellular stains.
(A) Wild type bone marrow was stained forCD19, CD43 and IgM. Cells were then fixed and permeabilized and stained with antibodies for the indicated intracellular proteins or the appropriate controls. Cells were gated for $CD19^{+ve}CD43^{-ve}IgM^{-ve}$ (pre-B), and the staining intensities of the indicated proteins plotted. For Pax5, Irf4, Ikaros and Aiolos unstained cells and control antibodies gave similar signals while the anti-protein antibody gave a higher signal. For Irf8, the control antibody did give a slightly higher signal compared to unstained cells however not as high as seen to the Irf8 antibody.
(B) HEK-293 cells were transfected with either GFP, YFP or the indicated fluorescent protein tagged proteins. Cells were then fixed, permeabilized and stained as indicated. For analysis cells were divided into positive and negative gates for the fluorescent proteins. For the Pax5, Irf4, Irf8, Ikaros and Aiolos antibodies, the cells gated as positive for the fluorescent-tagged proteins gave a higher signal than seen for either the cells that were gated negative for the transfected proteins or the GFP/YFP only transfected cells. This indicating that the antibodies were able to recognize the appropriate transfected proteins. (C) Details of the antibodies used for the intracellular stains are given and transfected proteins are given.
